# Tissue perfusion modelling in optical coherence tomography

**DOI:** 10.1186/s12938-017-0320-4

**Published:** 2017-02-08

**Authors:** Petra Stohanzlova, Radim Kolar

**Affiliations:** Department of Biomedical Engineering, Faculty of Electrical Engineering and Communication, Brno University of Technology, Technicka 12, 61600 Brno, Czech Republic

**Keywords:** Optical coherence tomography, Perfusion analysis, Deconvolution, Model, Phantom, Impulse response

## Abstract

**Background:**

Optical coherence tomography (OCT) is a well established imaging technique with different applications in preclinical research and clinical practice. The main potential for its application lies in the possibility of noninvasively performing “optical biopsy”. Nevertheless, functional OCT imaging is also developing, in which perfusion imaging is an important approach in tissue function study. In spite of its great potential in preclinical research, advanced perfusion imaging using OCT has not been studied. Perfusion analysis is based on administration of a contrast agent (nanoparticles in the case of OCT) into the bloodstream, where during time it specifically changes the image contrast. Through analysing the concentration-intensity curves we are then able to find out further information about the examined tissue.

**Methods:**

We have designed and manufactured a tissue mimicking phantom that provides the possibility of measuring dilution curves in OCT sequence with flow rates 200, 500, 1000 and 2000 μL/min. The methodology comprised of using bolus of 50 μL of gold nanorods as a contrast agent (with flow rate 5000 μL/min) and continuous imaging by an OCT system. After data acquisition, dilution curves were extracted from OCT intensity images and were subjected to a deconvolution method using an input–output system description. The aim of this was to obtain impulse response characteristics for our model phantom within the tissue mimicking environment. Four mathematical tissue models were used and compared: exponential, gamma, lagged and LDRW.

**Results:**

We have shown that every model has a linearly dependent parameter on flow ($R^2$ values from 0.4914 to 0.9996). We have also shown that using different models can lead to a better understanding of the examined model or tissue. The lagged model surpassed other models in terms of the minimisation criterion and $R^2$ value.

**Conclusions:**

We used a tissue mimicking phantom in our study and showed that OCT can be used for advanced perfusion analysis using mathematical model and deconvolution approach. The lagged model with three parameters is the most appropriate model. Nevertheless, further research have to be performed, particularly with real tissue.

**Electronic supplementary material:**

The online version of this article (doi:10.1186/s12938-017-0320-4) contains supplementary material, which is available to authorized users.

## Background

Optical coherence tomography (OCT) is a well established imaging technique used in different fields of clinical medicine, preclinical research or biology. The main capability of this technique is its ability to noninvasively create images of tissue on a micrometre scale and to perform so-called “optical biopsy”. During this process, information about tissue can be obtained from imaging. Several functional OCT techniques have been developed during the last decade, including Doppler OCT, polarisation-sensitive OCT and spectroscopic OCT. These techniques examine different properties of tissue and/or blood. In this study we describe a new application of functional OCT in the field of perfusion imaging, using nanoparticles as a contrast agent.

In general, perfusion is the process of delivering blood to capillaries in examined biological tissues. Perfusion analysis can be classified as a method of functional imaging and it forms an important part of the diagnosis of many diseases. Tissue perfusion can be evaluated by various modalities, including nuclear magnetic resonance (NMR), computed tomography (CT), positron emission tomography (PET) and ultrasound tomography. Conventional methods of perfusion analysis utilise the administration of a contrast agent into the bloodstream, where it specifically changes the image contrast. It is thus possible to monitor the amount (i.e. concentration) of contrast agent in a tissue that is supplied by a feeding artery.

Generally, it is possible to divide contrast agents into categories according to their behaviour. Intravascular contrast agents remain in the bloodstream, whereas extravascular contrast agents pass through the capillary walls into the extracellular space. For example, basic NMR brain perfusion models operate solely with intravascular contrast agents [1]. This is due to the impermeability of the blood–brain barrier [2]. Functional CTs generally operate with extravascular contrast agents and analyse the distribution of contrast in blood vessels and extravascular space of the tissue [3]. In the case of OCT, contrast agents may be intravascular as well as extravascular. This depends on their size, surface charge, and on the pore size of the tumour capillary endothelium [4].

It has recently been stated by Park [5] that “transport of nanoparticles after they have extravagated through tumour blood vessels has not been sufficiently described”. This transport has been described in different applications. For example, Aktas et al. [6] used modified chitosan-polyethylene glycol nanospheres to overcome a blood–brain barrier. Polymeric-based nanoparticles have recently received attention as a promising carrier for brain targeting [7, 8]. Lee et al. [9] found that single silver nanoparticles (5–46 nm) can be transported into and out of a zebrafish embryo through chorion membrane. In tissue bioreactors, the penetration of nanoparticles through the porous wall of the fibre can be used for controlled cell behaviour. This works particularly well in the application of magnetic forces to drive nanoparticles—for example, when fabricating fibrin gel with the appropriate nanostructure [10]. In such applications, advanced perfusion analysis can be used in order to obtain relevant information about perfused tissue/environment properties.

The development of every imaging modality is bound to phantoms simulating specific properties of living tissue. OCT phantom constructions vary from a simple water-based form, where it is possible to tune optical properties by adding a scattering substances (metallic particles, real blood cells, lipid emulsion etc.), [11] to a multilayer phantom with an embedded capillary system [12]. In our previous papers [13, 14], we presented single fibre perfusion phantom and proved the potential of the application of dilution theory in OCT modality. Here we describe a new phantom setup, which is convenient for perfusion analysis.

**Fig. 1:**
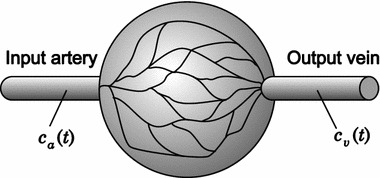
Physiological model of tissue blood supply. A simple model used in our experiment considers one feeding artery and one output artery, where the concentration on the contrast agent is measured

**Fig. 2:**
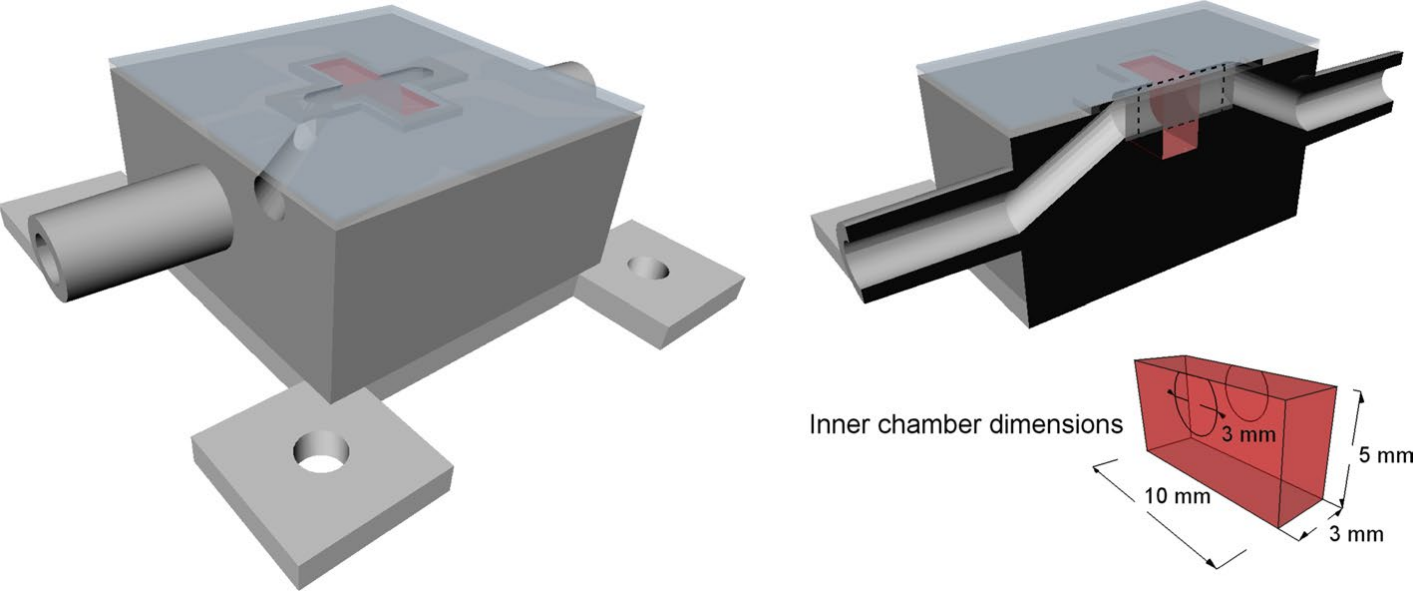
3D model of presented phantom. The inner chamber is filled with a NanobioMatrix Scaffold Sheet, represented in the figure by the *colour red* for better visibility. The *top right* figure shows the cut of the phantom model where the OCT tomographic ROI is illustrated by a *dashed line*. The *bottom right* figure shows the dimensions of the inner chamber; the diameter of input and output tubes is depicted as well

**Fig. 3:**
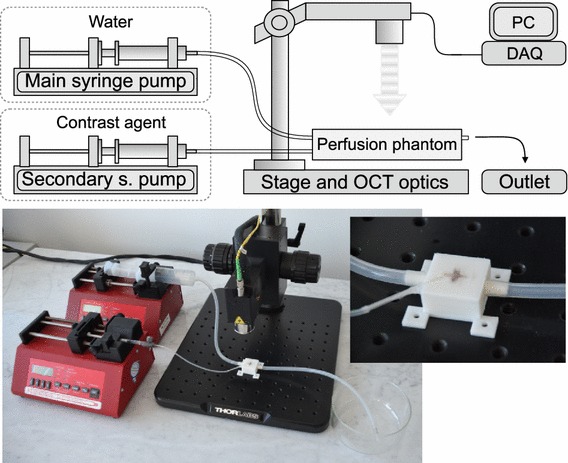
Principal scheme of experiment setup. The main syringe pump ensures a constant flow during a single experiment. The secondary syringe pump is used for fast bolus administration. The perfusion phantom is no-circulating, placed at the main OCT stage. The OCT system transfers data via a data acquisition (DAQ) board into the PC. A photography shows actual experiment setup and detail of the phantom

**Fig. 4:**
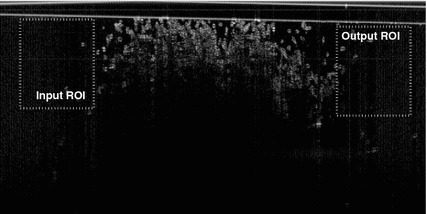
OCT intensity image with selected ROIs. The whole image corresponds to a *dashed rectangle* in Fig. 3. The inner chamber of the phantom presented is filled with a NanobioMatrix Scaffold Sheet, visible in the *middle part* of the image between the marked ROIs

**Fig. 5:**
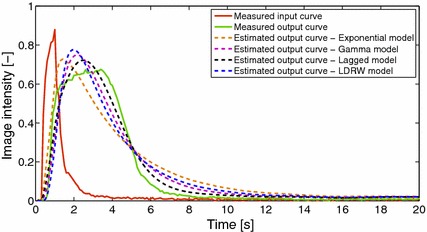
Example of dilution curves for nanorods A12N-10-1400 and flow rate 1000 μL/min. *Solid lines* illustrate measured dilution curves (input and output), while *dashed lines* depict the estimated output curves using four different models. The lagged model provides the best results in terms of fitting the shape of the curve. Results for other flow rates are in Additional file 1: Figure S1, Additional file 2: Figure S2, Additional file 3: Figure S3, Additional file 4: Figure S4, Additional file 5: Figure S5, Additional file 6: Figure S6, Additional file 7: Figure S7, Additional file 8: Figure S8

**Fig. 6:**
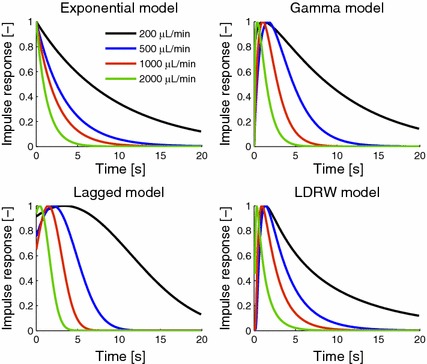
Impulse responses for nanorods A12N-10-1400, all tested models and flow rates. This figure illustrates the dependency of impulse responses on the flow rate for each model. Low flow rates correspond to a slower contrast agent washing out, resulting in a wider impulse response

**Fig. 7:**
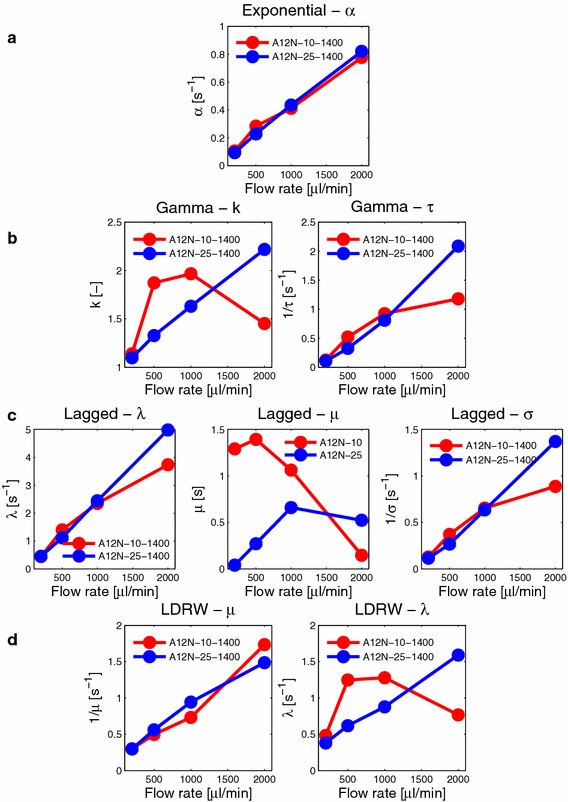
Parameter values and their dependency on flow rate. The *red line* is for nanorods A12N-10-1400 and *blue* for greater nanorods A12N-25-1400. At least one parameter of each model exhibits a dependency on the flow rate (**a** exponencial model, **b** gamma model, **c** Lagged model, **d** LDRW model)

The goal of this study is to apply and test basic perfusion theory to OCT modality. To our knowledge, this has not previously been applied. For this purpose, a simple perfusion tissue mimicking phantom has been created and a bolus-based perfusion approach has been applied during OCT scanning. The acquired images were processed by methods that use tissue convolution models. A similar setup is used in many preclinical research fields, for example, in general organ perfusion, and stroke or cancer research. Animal models are being used in these applications, particularly in cancer research. Tumour perfusion is influenced by the formation of new blood vessels within a tumour. This development of new vessels (e.g. angiogenesis) changes the image intensity and contrast of the tumour during an imaging procedure, when a contrast agent is employed that is specific to the imaging modality being used. OCT has only been used for the purpose of increasing tumour contrast without any advanced analysis (see i.e. [15, 16]) in spite of its availability and low cost in comparison to other imaging modalities (CT, NMR, PET or ultrasound). The advantages of OCT imaging are obvious—it is low cost, easy to use, and provides a sufficient spatial and temporal resolution for these kind of applications.

This paper is organised as followed. “Methods” section describes the convolution model, with different tissue impulse response functions. This is used on data acquired from the tissue mimicking phantom model (that will also be described in this section). The nanoparticles with two different sizes are used as a contrast agent. “Results and discussion” sections discuss the main findings of the deconvolution analysis. Finally, this study closes with some concluding remarks.

## Methods

### Perfusion analysis

The basic model of tissue blood supply is shown in Fig. 1—$c_{a}(t)$ is concentration in the input artery [also referred to as arterial input function (AIF)] and $c_{v}(t)$ is concentration in the output vein. In indicator dilution theory, tissues are described as a “black box”, without making any assumptions on transport processes and internal structures. Thus, contrast agent concentrations $c_{a}(t)$ and $c_{v}(t)$ are related by a convolution

1 $$\begin{aligned} c_{v}(t) = c_{a}(t) *q(t), \end{aligned}$$

where *q*(*t*) is the impulse response function characterising the system, contrast agent and interactions.

If $c_{a}(t)$ and $c_{v}(t)$ are known (measured), the impulse response function *q*(*t*) can be calculated by deconvolution methods.

In this paper, we have applied an approach where the impulse response function is modelled and estimated by optimisation techniques. Applied models of impulse response are described below. From the input concentration curve $c_{a}(t)$ and modelled impulse response function *q*(*t*), the output concentration curve $c_{v,calc}(t)=c_{a}(t)*q(t)$ is calculated. The impulse response model parameters are adjusted during minimisation in order to minimise the residual function [17]

2 $$\begin{aligned} \underset{\mathbf{p }}{\min }\sum _{t}\left| c_{a}(t)*q(t,\mathbf p )-c_{v}(t)\right| ^2, \end{aligned}$$

where $c_{a}(t)$, $c_{v}(t)$, mentioned above, are measured input and output concentration curves, $q(t,\mathbf p )$ is estimated impulse response function and $\mathbf p$ is a vector of its adjustable parameters. These have to be estimated during optimisation.

### Models

Applied models cover different types of approaches used for tissue perfusion modelling. The exponential and gamma models are compartment models, where the modelled system is considered as a series of compartments describing specific parts of a system. The lagged model belongs to mathematical models based on their similarity, with some specific mathematical function employed for data fitting. The category of physical models represents the local density random walk (LDRW) model, taking into account the physical behaviour of the contrast particles.

#### Exponential model

This model is the simplest type of compartment models. It consists of the single mixing compartment and it is described by the exponential function [17].

3 $$\begin{aligned} f_{Exp}(t) = \text {AUC}\alpha e^{-t\alpha }, \end{aligned}$$

where $c_{0}$ is the contrast agent concentration at time $t = 0$, and $\tau = 1/\alpha$ is a time constant depending on the flow rate and the compartment volume. AUC represents the area under the curve. It is a common parameter for all presented models and we have modified their representation to contain AUC. This allows for easier comparability.

#### Gamma model

The second compartment model is derived from the Erlang model [18], which is based on the assumption that constant blood flow can be modelled as a series of *k* mixing homogeneous compartments. If the constraint of an integer number of compartments is relaxed, we obtain Gamma model given by [19, 20].

4 $$\begin{aligned} f_{Gamma}(t)=\text {AUC}\frac{e^{-\frac{t}{\tau }}\left( \frac{t}{\tau } \right) ^{k-1}}{\tau \Gamma (k)}, \end{aligned}$$

where $\Gamma (k)$ represents the Gamma function.

#### Lagged model

In general, the lagged model is derived as a convolution of Gaussian function with one or more exponentials. The simple form of the lagged model can also be considered as a compartment model with two compartments—where the first compartment is a large vessel characterised by a Gaussian dispersion and the second is a microvascular bed which is a homogeneous mixing compartment described by a single exponential function [18]. The convolution integral has the form

5 $$\begin{aligned} f_{Lagged}(t) = \int _{-\infty }^{t}f(\tau ) g(t-\tau )d\tau , \end{aligned}$$

where

6 $$\begin{aligned} f(t) &= {} \frac{1}{\sqrt{2\pi \sigma ^{2}}}e^{-(t-\mu )^{2}/(2\sigma ^{2})},\quad-\infty <t <\infty , \nonumber \\ g(t) &= {} \, \lambda e^{-\lambda t},\,\quad t \ge 0. \end{aligned}$$

The lagged model can thus be expressed as

7 $$\begin{aligned} f_{lagged}(t) = \frac{\text {AUC}}{2}K\left[ 1+\text {erf}(L)\right] , \end{aligned}$$

where

8 $$\begin{aligned} K &= \lambda e^{-\lambda t + \lambda \mu + \frac{1}{2}\lambda ^{2}\sigma ^{2}}, \nonumber \\ L &= \frac{t-\mu -\lambda \sigma ^{2}}{\sqrt{2\sigma ^{2}}} \end{aligned}$$

and erf() is the Error function.

The parameter $\lambda$ represents a rate constant of exponential mixing compartment and parameters $\mu$ and $\sigma ^{2}$ are the mean transit time and the transit time variance of the compartment represented by the Gaussian distribution, respectively.

#### LDRW model

Diffusion with drift models describes the movement of indicator particles which is regarded as a longitudinal diffusion superimposed on a linear convection [18]. The LDRW model is

9 $$\begin{aligned} f_{LDRW}(t) = \frac{\text {AUC}e^{\lambda }}{\mu } \sqrt{\frac{\mu }{t} \frac{\lambda }{2\pi }} e^{-\frac{1}{2}\lambda (\frac{\mu }{t}+\frac{t}{\mu })}, \end{aligned}$$

where $\lambda =\text {Pe}/2$. $\text {Pe}$ is a Peclet number equal to the ratio between convection and diffusion in the dilution system [18]. Further, $\lambda ^{-1}$ is the skewness or asymmetry of the curve. The parameter $\mu$ is the transit time of the median indicator particle [21].

### Tissue phantom

The phantom used in this study is a specially shaped chamber produced by a 3D printer (Felix 3.1 dual extruder, FELIXrobotics). Polylactid acid (PLA) was used for printing, with 0.1 mm layer thickness. The phantom structure is shown in Fig. 2 and it was designed to match the physiological model of the tissue blood supply that is used in perfusion imaging (Fig. 1). The main dimensions of the phantom body (without fixing pads) are $25 \times 25 \times 13.5$ mm. The inner chamber is filled with NanobioMatrix Scaffold Sheet (PCL—Polycaprolactone, random fibres, thickness 3 mm; Synthecon, inc., Houston) that mimics a perfused tissue, its dimensions are $10 \times 3 \times 5$ mm. Two tubes lead to this chamber, simulating the input and output vessels (Fig. 1). An additional tube connected to the input tube is designed to deliver a bolus of contrast agent. The top is covered with a coverslip glued by transparent silicone sealant.

### Contrast agents

As a contrast agent in this experiment, two types of gold nanorods were utilised. Gold nanorods (Nanopartz Inc., A12N-10-1400 and A12N-25-1400) are in a form of colloidal suspension with water. Their parameters are summarised in Table 1. Both types have plasmon-resonant peak (1400 nm) matching the wavelength of our OCT system. Generally, gold nanorods are widely used for various biomedical applications, from imaging to therapeutical applications. They are very promising due to their tuneable optical properties and good biocompatibility [22–24]. Two sizes were used, because we want to simulate their different behaviours (i.e. perfusion) in our experimental setup. This could also be simulated by different properties of the scaffold sheets, but these were not available.

**Table 1:** Properties of gold nanorods, Nanopartz Inc.

Nanorods type	Plasmon-resonant peak (nm)	Diameter (nm)	Length (nm)	Concentration (mg/mL)
A12N-10-1400	1400	10	102	0.035
A12N-25-1400	1400	25	245	0.05

### OCT system

For this experiment, a Swept Source OCT system (Thorlabs, OCS1300SS, centre wavelength 1325 nm, other parameters are summarised in Table 2) was employed. Details about this OCT jtype and its advantages can be found in [25]. Our system provides RAW measurement data that was exploited in further processing and analysis. In the experiment presented, the acquisition rate was 10 images per second, proportions of the imaged area were set to the following values: image width 6 mm, image depth 3 mm and image resolution 1024 × 512 pixels.

**Table 2:** Parameters of Thorlabs system, OCS1300SS

Parameter	Value	Parameter	Value
Central wavelength	1325 nm	Coherence length	6.0 mm
Maximal imaging size	4000 × 512 pixels	Maximal volume size	$10\times 10\times 3$ mm
Spectral Bandwidth (FWHM)	>100 nm	Average output power	10 mW
Maximal imaging width	10 mm	Transverse resolution	$25\,$μm
Axial scan rate	16 kHz	Sensitivity	100 dB
Maximal imaging depth	3.0 mm	Axial resolution	$12/9\,$μm (air/water)

### Measurement methodology

The principal scheme of the experiment setup is depicted in Fig. 3. The phantom is connected with two syringe pumps (New Era NE-1010) by a silicon tubing system. While the first syringe pump ensures a constant flow through the phantom, the second syringe pump provides a bolus of contrast agent.

The experiment procedure itself consists of three phases. In the first phase, the constant flow rate (200, 500, 1000, 2000 μL/min) through the phantom is set by the main syringe pump. After this, the second syringe pump is activated with the flow rate 5000 μL/min to generate a bolus of contrast agent. The bolus volume is 50 μL. In the third phase, the same constant flow rate (200–2000 μL/min) continues until the end of the experiment. All these three phases are continuously imaged by the OCT system in one cross-section of the phantom (the plane of cross-section is illustrated in Fig. 2). The output is an image sequence with a rate of 10 images per second.

The procedure described was employed three times for each flow rate, within the above mentioned range, and with two types of gold nanorods.

### Data processing

Raw interference data produced by the OCT system without any processing has been used for processing in MATLAB (version R2012b; Optimisation, Curve Fitting and Image Processing Toolbox). An example of one intensity image from the sequence is in Fig. 4. There are two regions of interest (ROI) outlined in the image. The first indicates the area from where the input dilution curve is calculated, and the second corresponds to output dilution curve. ROIs were chosen in places right before and after the area, which corresponds to the matrix scaffold sheet, dimensions of both ROIs are approximately $170 \times 200$ pixels ($1.00 \times 1.17$ mm). The idea was to describe the tissue mimicking sheet. This was done by perfusion analysis performed on the data obtained from the areas closest to the material, but not inside it. The ROIs are also closest to the surface to eliminate the influence of attenuation.

The dilution curve is calculated as a median of pixels in ROI. Thus, it corresponds to median signal intensity. Figure 5 depicts an example of a dilution curve. The idea for median value extraction came from one of the speckle reduction methods [26]. The deconvolution method was applied to the measured curves from input and corresponding output ROIs. The goal of this procedure was to compare different models of impulse response *q*(*t*), and to found the best model for producing computed output curves $c_{v,calc}(t)$ that correctly fit the measured curves.

To solve the deconvolution problem of unconstrained nonlinear optimisation, the simplex search method [27] was applied. It is a direct search method that uses only function values, without any derivative information. The initial values of model parameters were randomly selected from ranges listed in Table 3. The minimisation was repeated 20 times to eliminate problems with the local maximum. The best result of the model parameters was chosen according to its lowest criterion value (Eq. 2).

**Table 3:** Parameters initial values

Model	Parameter	Initial value range
Exponential	$\alpha$	0.1 to 1 s$^{-1}$
Gamma	*k*	0.1 to 3
	$\tau$	0.1 to 3 s
Lagged	$\lambda$	0.1 to 5 s$^{-1}$
	$\mu$	0.1 to 2 s
	$\sigma$	0.1 to 2 s
LDRW	$\mu$	0.1 to 5 s
	$\lambda$	0.1 to 5

The range for each model has been determined by analysis of the influence of each parameter on the shape, taking into account the temporal properties of measured concentration curves

All models and their parameters were analysed with respect to their dependency on the flow rate. Results are presented in the next section.

## Results and discussion

The results of the impulse response optimisation are evaluated and discussed in the subsection bellow. The relation between model parameters and flow rates is analysed and discussed in the next subsection. The influence of both nanorods is also discussed.

### Optimization results

As an example, Fig. 5 illustrates the dilution curves measured and calculated for flow rate 1000 μL/min and all the models that have been applied. The input curve is represented by solid red, the measured output curve is illustrated as a solid green line, and the modelled and calculated output curves have dashed lines.

As can be seen, the fitting is not perfect, but the results are different for different models and different values of flow rates. The best results, with respect to the optimisation criterion, were achieved with the lagged model in the entire range of the applied flow rates (Table 4). The values in the brackets represent a variation of the minimisation criterion for 20 random repetitions with different initialisations.

**Table 4:** The best values of minimisation criterion for different models and flow rates

	Flow rate (μL/min)			
	200	500	1000	2000
A12N-10-1400				
Model				
Exponential	1.78 (±0.00)	1.27 (±0.00)	1.18 (±0.00)	0.35 (±0.00)
Gamma	1.75 (±0.00)	1.08 (±0.00)	0.79 (±0.00)	0.27 (±0.00)
Lagged	*1.27* (±*3.90)*	*0.65* (±*0.79)*	*0.51* (±*0.38*)	*0.19* (±*0.35*)
LDRW	1.35 (±0.00)	1.25 (±0.00)	1.00 (±0.00)	0.34 (±0.00)
A12N-25-1400				
Model				
Exponential	9.21 (±0.00)	4.97 (±0.00)	3.37 (±0.00)	2.68 (±0.00)
Gamma	9.20 (±0.00)	4.56 (±0.00)	2.59 (±0.00)	1.70 (±0.00)
Lagged	*8.18* (±*11.25)*	*3.51* (±*2.91)*	*1.78* (±*0.84)*	*1.35* (±*0.22)*
LDRW	10.75 (±0.00)	5.66 (±0.00)	3.30 (±0.00)	1.99 (±0.00)

The number in brackets represents the variability of optimisation results. If the value is 0.00, the optimisation resulted with identical solution in each repetition

The best result is highlighted by italic

It can be seen that criterion value is higher for lower flow rates, which implies that selected models and methodology is more convenient for higher flow rates. We also observed that the shape of the output curves is usually fitted better in ascending part than descending part. Another problematic part is fitting the shape around maximum of the curves.

The optimisation results were also evaluated by $R^2$ values, which measure the goodness of fit (Table 5). This value has been calculated between $c_{v,calc}$ and $c_{v,meas}$. The highest values (the best fit) has achieved a lagged model.

Another model comparison was done from the point of view of perfusion parameters. We compared two perfusion parameters AUC and MTT (mean transit time), again obtained from $c_{v,calc}$ and $c_{v,meas}$. Results are shown in Table 5. The italic letters represent values, which are closer to the measured values (also italic). The lagged model achieved the best results up to the AUC value for flow rate 1000 μL/min. This implies the superiority of the lagged model over the other tested models. The only disadvantage of this model is its dependence on the initial parameter values, which is probably caused by three model parameters (other models have one or two parameters).

**Table 5:** Goodness of fit characterised by *R* ^2^ values, mean transit time (MTT) and area under the curve (AUC) for each model and flow rate (μL/min)

Flow rate	Exponential	Gamma	Lagged	LDRW	Measured
R^2^					
200	0.919	0.922	*0.939*	0.885	–
500	0.884	0.943	*0.973*	0.907	–
1000	0.863	0.932	*0.965*	0.898	–
2000	0.860	0.879	*0.906*	0.848	–
MTT					
200	14.93	14.93	*13.78*	15.59	*12.46*
500	8.41	8.15	*7.92*	8.35	*6.18*
1000	5.78	5.58	*5.45*	5.70	*3.74*
2000	3.27	3.19	*3.10*	3.27	*2.36*
AUC					
200	145.47	139.88	*136.56*	139.11	*134.57*
500	63.41	59.11	*58.06*	58.84	*56.23*
1000	34.09	32.53	32.66	*32.08*	*28.16*
2000	8.59	8.28	*8.14*	8.29	*7.58*

Italics values correspond to the best results. For $R^2$ it is the highest value. For AUC and MTT it is the value closest to measured curve value

### Parameters of models

Modelled impulse response functions for the models tested are illustrated in Fig. 6 (nanorods A12N-10-1400; the median value from three measurements has been used for plotting this image, see below). As can be seen, the width of the impulse response curves depends on the flow rate; for lower flow rates a higher width of impulse responses can be observed. These curves represent how, if an ideal bolus is applied, the tissue mimicking model transfers the (concentration of) nanoparticles.

Further, we examined the relation between model parameters, flow rates and nanoparticle size (see Fig. 7). In every figure, the median value of three performed measurements is shown. The models used in our analysis have different parameters, but the parameter related to flow can be found in every model. The next parameters of specific models refer to some other physical property of the tissue mimicking environment.

The only parameter of the exponential model is $\alpha$ and influences the width of the curve, implying its dependency on the flow rate. From Fig. 7a, this linear trend is evident. This increasing tendency is consistent with the assumptions, because an increase in the value of the exponent causes a narrowing of the curve. There is no statistical difference between nanorods types.

The gamma model has two parameters $\tau$ and *k*. Parameter $\tau$ is inversely proportional to the parameter $\alpha$ of the exponential model, and it also has a dependency on the flow rate. Thus, its reciprocal value is displayed in Fig. 7b for easier comparison of these two models. The linearity can be also seen for $\tau$ like in an exponential model, with saturation for small nanoparticles and higher flow rates. Parameter *k* expresses the number of compartments. In our case, parameter *k* assumes values in the range approximately 1–2.5, which is adequate for the presented model. The main compartment is the tissue model. The influence of the second compartment can be interpreted as a nanoparticle mixing in the volume where the bolus is injected (the input artery). For higher flow rates, higher mixing with tendency to turbulence can be expected. For smaller particles, the *k* value decreases with a higher flow rate; this implies that the whole system behaves as a single compartment.

The first parameter of the lagged model is a parameter $\lambda$ that represents the rate constant of the exponential mixing compartment; therefore it is comparable with the rate constant in the exponential model. Thus, its dependency on flow rate is very similar to previous models (Fig. 7c). The second two parameters $\mu$ and $\sigma$ relate to the Gaussian compartment, representing the mixing before the tissue model. Parameter $\sigma$ influences the width of the curve and consequently it is dependent on the flow rate. Higher sigma values correspond to slower mixing for low flow rates, and vice versa. Parameter $\mu$ has the meaning of Gaussian mean value and determines the position of the curve peak. The range of $\mu$ values is almost from zero to 1.4 s, which is very small in comparison to experiment duration. Also, no recognisable dependence on the flow rate can be seen. Therefore, we can consider this parameter as insignificant.

The model tested last is the LDRW model with parameters $\mu$ and $\lambda$. The parameter $\mu$ is also linearly dependent on the flow rate (Fig. 7d) and can be compared with the corresponding flow rate-related parameters from other models. The parameter $\lambda$ provides an indication of the relative importance of diffusion and convection. It should increase with increasing flow rates as the convection dominates. This can be only seen for the larger nanoparticles. The curve for smaller nanoparticles shows saturation and small decrease. This is related to changes in convection/diffusion influences that are due to turbulence. Figure 7 reveals an interesting correspondence between the parameter $\lambda$ from the LDRW model and parameter *k* from the gamma model, representing the number of compartments. As $\lambda _{\text {LDRW}}$ is proportional to the Peclet number, this correspondence with $k_{\text {Gamma}}$ indicates that diffusion prevails over convection.

We have shown that, irrespective of the model, the flow-related parameter has a linear relation to flow, which has been tested by linear regression and $R^2$ values. This had the range 0.4914–0.9996, with the best results for the exponential and lagged model, and the worst for the gamma model. This parameter can therefore be used as a flow indicator in bolus-based perfusion analysis. Furthermore, the gamma model provides parameter *k* (non-integer number of compartments), which can be used for characterising, or for better understanding the examined tissue. The lagged model provides the best fits over all flow rates, with respect to the minimisation criterion (see Table 4). The LDRW model can help us to better understand the physical processes connected to diffusion/convection processes in capillaries.

The parameters of each model have some specific physical meaning. The flow-related parameter can be found in each model; therefore the tissue perfusion can be estimated when used in a real setting. The other parameters have different meanings of describing the tissue. Another two commonly used perfusion parameters (MTT, AUC) can also be obtained for the examined tissue. MTT is the mean time taken by blood to pass through the capillary network. These parameters depend on tissue perfusion and the volume of blood flow in the tissue. They are usually computed from the tissue concentration curve, which is influenced by AIF. This approach allows us to reduce the impact of AIF and to directly extract AUC and MTT from the estimated model functions.

## Conclusion

We have demonstrated a new application of OCT for perfusion analysis using a bolus-based approach. The results presented are based on a tissue mimicking model; a simple model of capillary tissue. The mimicking of tissue is of course limited. In a real tissue, different behaviour can be expected due to different interactions between nanoparticles, blood vessel walls and blood components. The used nanoparticles can be both, intravascular or extravascular/extracellular, depending mainly on their size and surface charge and also on pore size of (tumour) vessels [4]. Currently, in OCT, modified chitosan-polyethylene glycol nanospheres has been used to overcame a blood brain barrier [6]. The transport of silver nanoparticles (5–46 nm) into and out of the zebrafish embryo through chorion membrane has been observed using OCT [9]. These are examples of applications where the presented approach can be used to quantify examined tissues.

Application of our simple phantom is a first step in this quantitative OCT contrast imaging area. The basic principle of this deconvolution-based perfusion theory can be tested and evaluated using this setup. The properties of a nanofibre scaffold sheet allow us to use only limited values of flow rates, which can influence the optimisation and analysis of the model. However, the lagged model has three free parameters. This allow us to set different properties (or shapes), and therefore fit to different conditions. Interpreting the parameters is also relatively straightforward. We showed that each model contains parameters linearly related to flow rate. We used two types of nanoparticles in order to simulate different behaviours, but this has only been proved for the *k* parameter from the gamma model and the parameter $\lambda$ from the LDRW model. These two latter models are relatively complex and their parameters can describe, from different perspectives, the properties of tissue being examined.

One step that can be taken in the future is to test this approach on real tissue (e.g. tumour tissue on animal model). To make this method applicable in real preclinical, or even clinical settings (e.g. skin tumours), the attenuation problem must be solved. The whole signal attenuation consists of tissue attenuation and nanoparticles attenuation. Several methods on this topic have already been published [28, 29]. The recirculation of nanoparticles is another issue. Nevertheless, the single-pass can be approximate, or the convolution model can be more complex, in order to simulate the second pass. A final important issue is motion compensation, which occurs during animal breathing by image registration method.

## Additional files

**Additional file 1: Figure S1.** Dilution curves for nanorods A12N-10-1400 and flow rate 200 μL/min. Solid lines illustrate measured dilution curves (input and output), while dashed lines depict the estimated output curves using four different models. The lagged model provides the best results in terms of fitting the shape of the curve.

**Additional file 2: Figure S2.** Dilution curves for nanorods A12N-10-1400 and flow rate 500 μL/min. Solid lines illustrate measured dilution curves (input and output), while dashed lines depict the estimated output curves using four different models. The lagged model provides the best results in terms of fitting the shape of the curve.

**Additional file 3: Figure S3.** Dilution curves for nanorods A12N-10-1400 and flow rate 1000 μL/min. Solid lines illustrate measured dilution curves (input and output), while dashed lines depict the estimated output curves using four different models. The lagged model provides the best results in terms of fitting the shape of the curve.

**Additional file 4: Figure S4.** Dilution curves for nanorods A12N-10-1400 and flow rate 2000 μL/min. Solid lines illustrate measured dilution curves (input and output), while dashed lines depict the estimated output curves using four different models. The lagged model provides the best results in terms of fitting the shape of the curve.

**Additional file 5: Figure S5.** Dilution curves for nanorods A12N-25-1400 and flow rate 200 μL/min. Solid lines illustrate measured dilution curves (input and output), while dashed lines depict the estimated output curves using four different models. The lagged model provides the best results in terms of fitting the shape of the curve.

**Additional file 6: Figure S6.** Dilution curves for nanorods A12N-25-1400 and flow rate 500 μL/min. Solid lines illustrate measured dilution curves (input and output), while dashed lines depict the estimated output curves using four different models. The lagged model provides the best results in terms of fitting the shape of the curve.

**Additional file 7: Figure S7.** Dilution curves for nanorods A12N-25-1400 and flow rate 1000 μL/min. Solid lines illustrate measured dilution curves (input and output), while dashed lines depict the estimated output curves using four different models. The lagged model provides the best results in terms of fitting the shape of the curve.

**Additional file 8: Figure S8.** Dilution curves for nanorods A12N-25-1400 and flow rate 2000 μL/min. Solid lines illustrate measured dilution curves (input and output), while dashed lines depict the estimated output curves using four different models. The lagged model provides the best results in terms of fitting the shape of the curve.

## Abbreviations

OCT: optical coherence tomography
NMR: nuclear magnetic resonance
CT: computed tomography
PET: positron emission tomography
AIF: arterial input function
AUC: area under the curve
LDRW: local density random walk
PLA: polyactid acid
PCL: polycaprolactone
ROI: region of interest
MTT: mean transit time
